# Computational Modeling as a Tool to Drive the Development of a Novel, Chemical Device for Monitoring the Injured Brain and Body

**DOI:** 10.1021/acschemneuro.3c00063

**Published:** 2023-09-22

**Authors:** De-Shaine Murray, Laure Stickel, Martyn Boutelle

**Affiliations:** † Department of Bioengineering, Imperial College London SW7 2AZ, London, U.K.; ‡ School of Engineering and Applied Sciences, Yale University, 06520, New Haven, Connecticut United States; § Laboratoire Physico-Chimie Curie, Institut Curie, 26 rue d’Ulm, 75005, Paris, France

**Keywords:** neuromonitoring, computational modeling, microdialysis, neurotrauma, chemical, analytical

## Abstract

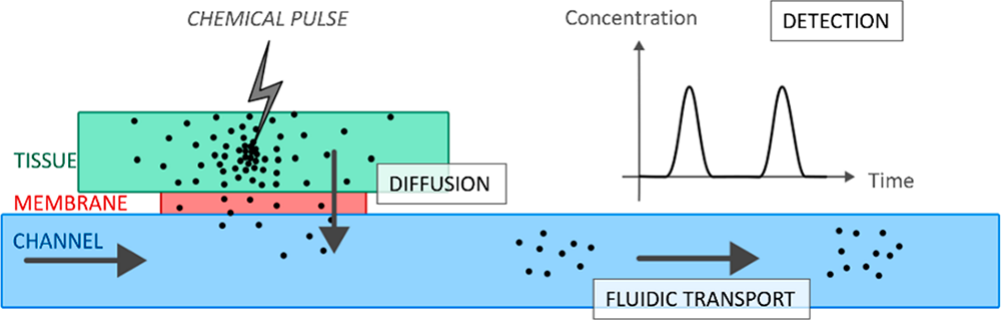

Real-time measurement of dynamic changes, occurring in the brain and other parts of the body, is useful for the detection and tracked progression of disease and injury. Chemical monitoring of such phenomena exists but is not commonplace, due to the penetrative nature of devices, the lack of continuous measurement, and the inflammatory responses that require pharmacological treatment to alleviate. Soft, flexible devices that more closely match the moduli and shape of monitored tissue and allow for surface microdialysis provide a viable alternative. Here, we show that computational modeling can be used to aid the development of such devices and highlight the considerations when developing a chemical monitoring probe in this way. These models pave the way for the development of a new class of chemical monitoring devices for monitoring neurotrauma, organs, and skin.

## Introduction

Analytical chemical monitoring devices are an important way of studying diseased and injured tissue. They typically involve a sampling element, often a commercial microdialysis probe, coupled to a microfluidic manifold that incorporates relevant chemical sensors. Devices of this nature are finding increasing utility due to improvements in real-time, continuous, chemical monitoring and advancements in sampling tissue and organs, such as the brain, by less invasive means. This paper describes the design and development, using the aid of computational modeling, of a new class of sampling device that incorporates tissue sampling directly into the sensor-containing manifold.

The successful sampling and monitoring of tissue extracellular fluid (ECF) can provide vital information for clinicians.^1^ Chemical levels that deviate from typical values in the blood and ECF can be useful indicators of the proliferation of disease, degeneration, and damage.^2^ The accepted standard for chemical monitoring is the sampling of blood, which requires the repeated removal of small samples for offline analysis using high performance liquid chromatography (HPLC) or mass spectrometry (MS). This analysis can be time-consuming and often misses dynamic changes that occur at faster time scales, for example during the acute progression of diseases.^3^

Accurate detection of patient deterioration by quickly elucidating patterns in disease and injury progression can be achieved by online, real-time measurement facilitated by integrated microfluidic channels within analytical chemical devices.^4^ Three main approaches can be employed to achieve continuous monitoring: (1) Implanted electrodes with modified surfaces for chemical transduction through biosensing;^5^ (2) optical devices that sense in close proximity to the media in question—using light sources;^6,7^ and (3) sampling devices such as microdialysis probes which deliver samples representative of ECF for ex-vivo analysis.^5^ Of these methods, implanted electrodes often suffer from disturbances of the electrode surface that lead to drift, ultimately reducing the measurement accuracy and precision over time.^8^

Conversely, optical methods, although noninvasive and thus the most desirable, often lack sufficient sensitivity to provide reliable reporting of dynamic changes in chemical concentrations.^9^ Therefore, implantable microdialysis probes that act as delivery devices for analytical equipment outside of the body provide a middle ground between the aforementioned methods.^10^ When microdialysis is coupled with microfluidics and linked to ex-vivo biosensors, continuous monitoring of dialysate can be achieved.^11^ A distinct advantage here is all sensing apparatus is found outside of the body and thus readily accessible and can be easily replaced when performance issues are observed.^12^ This has been shown with the development of continuous online microdialysis (coMD)^13,14^ which displays data at 200 samples per second, with a slight offset delay.

However, the probes that afford this analysis are concentric in nature and penetrate tissue directly to establish a diffusional concentration gradient. On the order of a few 100 μm, such diameters create penetration injuries, which lead to inflammation and the development of barriers of tissue that can surround and hamper the sampling probe.^15,16^ The retrodialysis of dexamethasone, a glucocorticoid anti-inflammatory, has been employed by Varner et al, to alleviate such barriers by reducing the proliferation of abnormal tissue and thus enhancing the level of detection at the sampling zone.^17,18^ However, such pharmacological interventions can be completely avoided by designing surface probes that do not have to be inserted into the cortex and as such do not create penetrative injuries upon placement. It is worth noting that such devices can only currently be utilized when a craniectomy or craniotomy has occurred but the “softness” of these prospective technologies means that surface probes could be folded and introduced in minimally invasive ways, such as through a burr hole, before being unraveled.^19,20^

Surface microdialysis (s-μD) is not a new concept. Foundational work from Abrahamsson, Akesson, and colleagues led to the development of a probe that can be sutured to the heart, bowel, liver, and other tissue.^21−23^ This device is now commercially available and is known as the OnZurf probe. The devices that follow the general principles described here build on this work but incorporate soft, flat, and flexible materials and a different form factor. Such materials allow for the incorporation of flexible electronics using soft lithography and the possibility of developing new surgical protocols to reduce the risk of surgery on a very fragile region of the body.^20^

By extension, such devices can also make for useful environments to support cell growth, monitoring, and the development of cellular layers into organs, for organ-on-a-chip (OOAC) and organ-in-a-chip (OIAC) applications. The use of semipermeable membranes can allow for the delivery of nutrients to a tissue chamber and the corresponding microfluidics can be used for rapid chemical stimulation.^24,25^ Therefore, the development of soft, flexible, near 2-dimensional sampling microdialysis probes solves many immediate issues with chemical monitoring of tissues and has a wide applicability that spans multiple subfields of bioengineering. Such devices open the possibility of monitoring tissues on different scales:

- In-vivo monitoring could be easily implemented to assess the brain after trauma, with thin, conformal and biocompatible devices being placed directly on the surface of the brain under the dura. Less damage would be sustained to the tissue during implantation, reducing the foreign body response (FBR) and increasing the viability and longevity of the implanted device.
- Ex-vivo monitoring would benefit from the use of such devices. For example, the chemical state of a kidney could be continually monitored for health and function in transit, without penetrative injuries.^26^ As transplant organs are very sensitive, preserving their integrity while getting clear updates about their health (with minimal damage) would be essential to performing successful transplant operations.
- OOAC and OIAC experiments could be developed using these devices where tissue slices could be placed in close proximity to, or be directly embedded within, the device. Using the microfluidic characteristics of the analytical chemical device, the conditions and delivery of nutrients these tissues would need to survive could be mimicked, in addition to the simulation of disease states.^27−29^
- Skin monitoring using such noninvasive, conformal devices could monitor the composition of sweat on the surface of the skin.^30^ Consumer health, such as fitness monitors or continuous glucose monitoring for diabetes, could benefit from such form factors.

Current fabrication methods offer great freedom of design; therefore, highlighting the critical parameters and design features for effective chemical sampling using microfluidics will greatly aid the development of efficient prototypes. One way of ascertaining these critical parameters is by constructing possible prototypes using computational models. Applying modeling to microfluidic geometries to assess the fluid dynamics and performance of a system is a quick way to home in on feasible, real-world solutions.^31^ Such modeling has already been utilized within this field to investigate drug delivery using retrodialysis and tissue damage from low-flow perfusion devices.^32−34^ Computational modeling is therefore becoming an increasingly useful tool that allows for the iterative evaluation, development, and optimization of microfluidic and microdialysis systems.^35^

One such computational modeling environment is COMSOL Multiphysics. This is an interactive environment that can be used for solving a range of scientific and engineering questions. COMSOL works on the basis that partial differential equations form the basis of fundamental scientific laws. Within the software, models can be built that simulate physics phenomena by combining these partial differential equations, without the need for an in-depth knowledge of mathematics. A key differentiator of COMSOL is the ability to model and investigate multiple phenomena at the same time, which is more indicative of real-world scenarios, where multiple variables can impact the performance of your system.^36^ However, users of such software should be aware that such tools should only be used as a guide for their experimental counterparts and are, more often than not, based on assumptions and simplifications. In addition, although time can be saved by using modeling such as COMSOL, there is a computational cost, and this increases with the complexity of the model that is created. With the increase in computational power available at a low cost, we can also go beyond modeling simple fluidic components as circuit analogies or numerical solutions and generate large data sets without conducting physical experiments. For even more complex systems, where a system cannot be adequately modified from first-principles, machine learning can be utilized to probe complex microfluidic behavior.^37,38^

In this paper, we show how modeling can be implemented, in order to optimize the prototyping of near-2D sampling devices. We consider the separation of consecutive signals, multiple sampling points, and changing the geometry of channels in order to ascertain key considerations for robust chemical sampling with high resolution and minimal time delay, with potential applicability to neuromonitoring.

## Results and Discussion

### Modeling Assumptions

The development of these models was predicated on a set of key assumptions: First, simulated cerebral spinal fluid (CSF), an in-silico representation of the medium in which such devices would be encompassed, has been modeled as water. The effects of the contents of CSF on diffusion are taken into account in the diffusion (*D*) value, and its viscosity is similar to water. Similarly, the neurochemical molecules of interest are considered as particles with interactions taken into account in the *D* value. The incompressibility and Newtonian behavior are reasonable assumptions for a fluid that behaves similarly to water. In terms of geometry, the walls have been considered perfectly parallel, which may differ from those of fabricated probes. However, since we mostly extract tendencies, not specific values for each parameter, the results are still informative for nonideal geometries. The diffusivity in the membrane has been modeled as isotropic; this is not the case for most real membranes; however, since the membrane is extremely thin (10 μm), the concentration gradient is always much higher in the cross-membrane direction rather than longitudinally inside. We can therefore assume that particle diffusion is mostly through the membrane. The concentration applied either directly on the channel (models without diffusion) or on top of the tissue (full model) was 10 mmol/L. Elsewhere, the concentration was 0 mmol/L.

### General Dynamics -2D Exploration

The first set of simulations was aimed at understanding the general dynamics of a microfabricated analytical chemical device and checking whether common intuition proved correct. All of these observations were based on 2D simulations, with a side or top view and the concentration applied directly on a wall of the channel. The pulses were very short (5 to 10 s) in order to have short computation times. One area of great interest to these devices is the effective surface area of the membrane exposed to the tissue. The idea that increasing this surface area could lead to a higher concentration being detected at the end of the channel was confirmed for short pulses. An increase in the membrane length visually increased the number of particles in the channel. However, the increase in membrane length does not dramatically increase the size of the diffusion pattern, which, in our parameter range, was found to be more directly influenced by the pulse length.

For microdialysis purposes, the membrane diffusion coefficient (*D*_m_) ranges from 10^–9^ to 10^–10^ m^2^/s. The thickness of the membrane was denoted as Mem_t_. *D*_m_ can be adjusted to match the molecular weight cut off (MWCO) or other properties of the membrane. The use of multiple modules, a benefit of COMSOL, allows for charge (both of the membrane and ionic species) or hydrophobicity/hydrophilicity to be included in a more complex model. The downward velocity from the tissue to the channel is therefore *D*_m_/Mem_t_, which means that for Mem_t_ ≈ 10 μm, the time that a particle takes to cross the membrane ranges from *t*_1_ = 10 ms to *t*_2_ = 100 ms. Both are almost instantaneous compared to other time scales in the model, so the membrane diffusion coefficient does not have a major influence on the particles that enter the channel and thus the concentration that is detected at the end of the channel. When the channel geometry is extremely small (10s of microns), the particles quickly reach their maximum concentration and diffusion is halted, which limits the entry of subsequent particles into the channel and thus the concentration detected at the sensors. On the opposite end, when the channel is too big, the particles are diluted within the channel and although the particles entering the channel are constant, the concentration detected at the end of the channel decreases. Finding an optimal range will be a critical parameter for the development of future devices.

### 3D Simulations—Reaching the Detection Threshold

**Figure 1:**
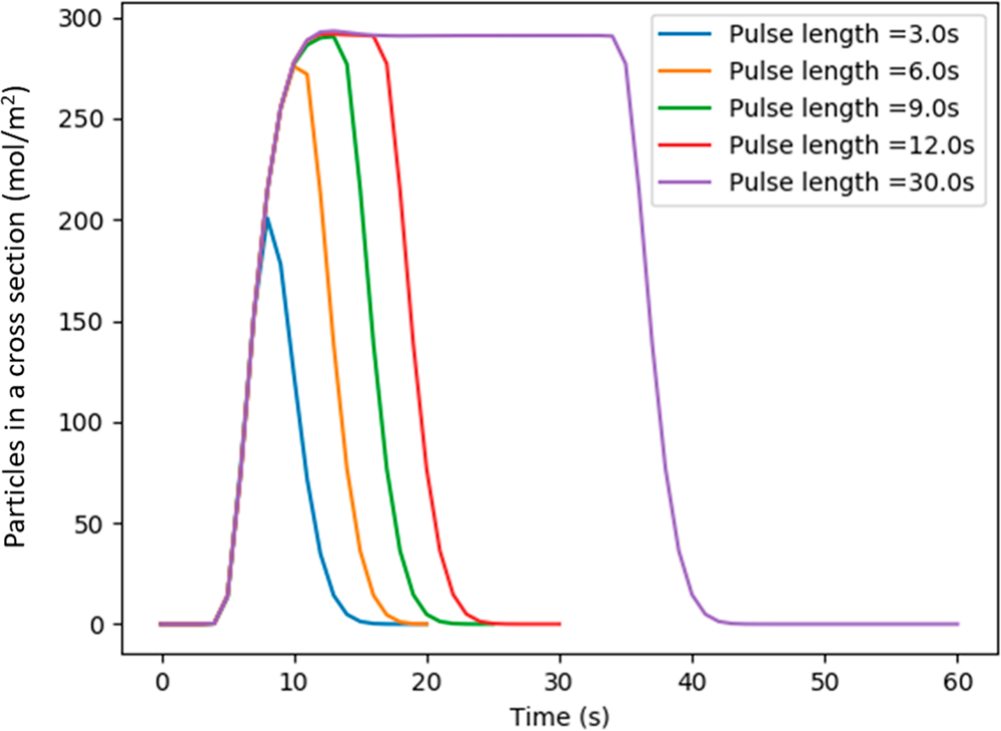
Pulse length variation for a typical set of parameters (Ch_h_ = 100 μm, Ch_w_ = 200 μm, Mem_w_ = 200 μm, *V* = 25 mm/min): the plateau is only reached for pulses longer than 9 s.

After validating the general dynamics of the system in 2 dimensions, 3-dimensional simulations were generated, and specific requirements could then be investigated. The first requirement of these microfabricated analytical chemical devices is being able to reach the detection levels of the sensors. When a chemical pulse is generated in the tissue, if the pulse length is too short, as a consequence of Taylor dispersion, the concentration detected in the channel follows approximately a Gaussian curve, and the maximum value is directly related to the pulse length, i.e., the temporal reactivity of the system. Above a critical time, the concentration in the channel reaches a plateau (Figure 1), and the aim is then to increase the height of the whole plateau, which does not depend on the temporal reactivity of the system but on its efficiency. Results from the model suggest that for this system with the specific set of parameters, pulses around 10 s in duration and above are not affected by the temporal reactivity of the system.

#### Flow Rate

**Figure 2:**
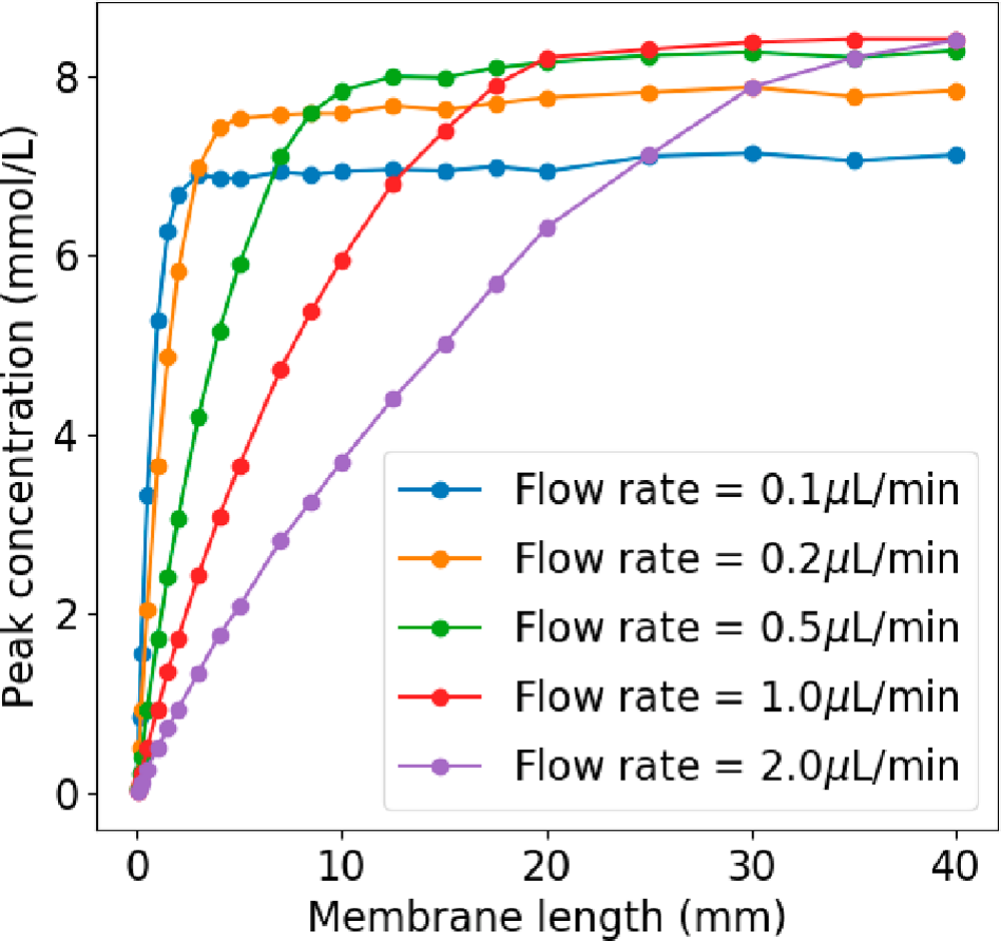
Concentration of particles detected for varying membrane lengths and flow rates. The geometry (Ch_h_ = 100 μm, Ch_w_ = 200 μm, Mem_w_ = 200 μm) is identical for each simulation. The variations of the flow rate are obtained by changing the fluid velocity.

The flow rate is a vital parameter in changing the characteristics of the concentration profile of the channel. Reducing the flow rate allows more time for particles to diffuse across the membrane, leading to a higher concentration in the dialysate. For a 5 mm-long membrane, reducing the flow rate by a factor of 10 can multiply the concentration detected at the end of the channel by almost 4 (Figure 2).

However, decreasing the flow rate also means that the average linear velocity perceived by the particles in the layer close to the membrane will be lower. Therefore, the cross-membrane concentration gradient will be less favorable to particle crossing.

As a result, for a given membrane size, the plateau limit for the concentration in the channel is slightly higher for faster flow rates: for a 20 mm-long membrane, the limit is 8 mmol/L for a flow rate of 0.5 μL/min and only 7 mmol/L for a flow rate of 0.1 μL/min. Results from the model suggest that the choice of flow rate can greatly influence the resulting concentration detected, where a reduction in the flow rate leads to an increase in the concentration that is sensed at the end of the channel. The choice of flow rate is also highly dependent on the membrane length, and the two parameters should be considered together.

#### Membrane Length

**Figure 3:**
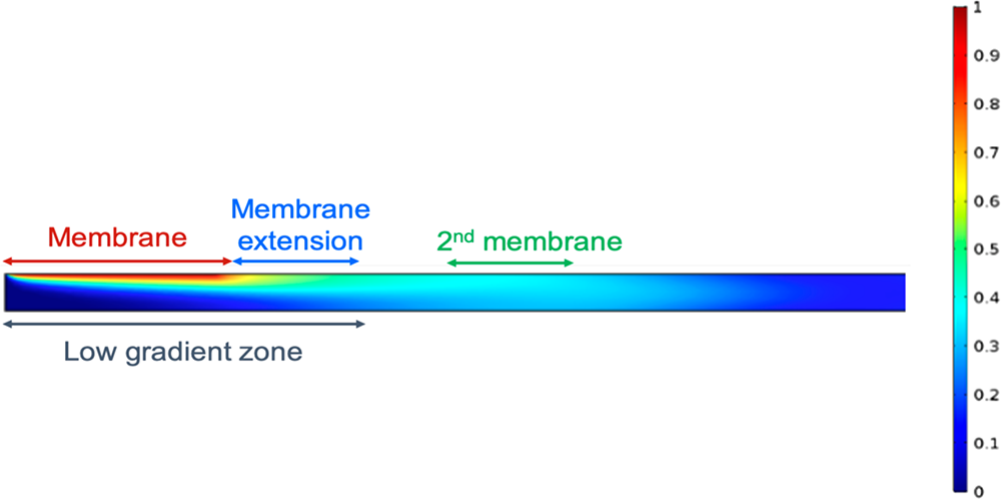
Simulation of the cross section of a channel to visualize the cross-membrane gradient. Ch_h_ = 100 μm.

Increasing membrane length is an efficient way for more particles to enter the channel, but the effect is not linear. Particles crossing the membrane are dragged by the fluid flow along the channel while they diffuse much more slowly toward the bottom, hence they accumulate in a diffusion layer close to the membrane (low gradient zone on Figure 3). Therefore, if we extend the membrane (membrane extension in Figure 3), the particles along the extension will face a cross-membrane gradient which would be lower than if the two membranes were independent. For a given membrane area, it would therefore be more efficient to place the extension further away (2nd membrane on Figure 3), where the gradient is more favorable to particle diffusion. It must be noted that such a change would be at the expense of spatial resolution. Figure 2 shows that at some point, the concentration reaches a plateau even if the membrane gets longer because the whole layer close to the membrane is saturated. Results from the model suggest that having a longer membrane length increases the number of particles that enter the channel and are detected at the end of the channel but an even greater efficiency can be achieved by dividing the membrane into segments, which would alleviate saturation and facilitate more particle diffusion. This would be a useful design to employ if spatial resolution is not a concern and only one measurement is being taken from the probe.

#### Channel Width

**Figure 4:**
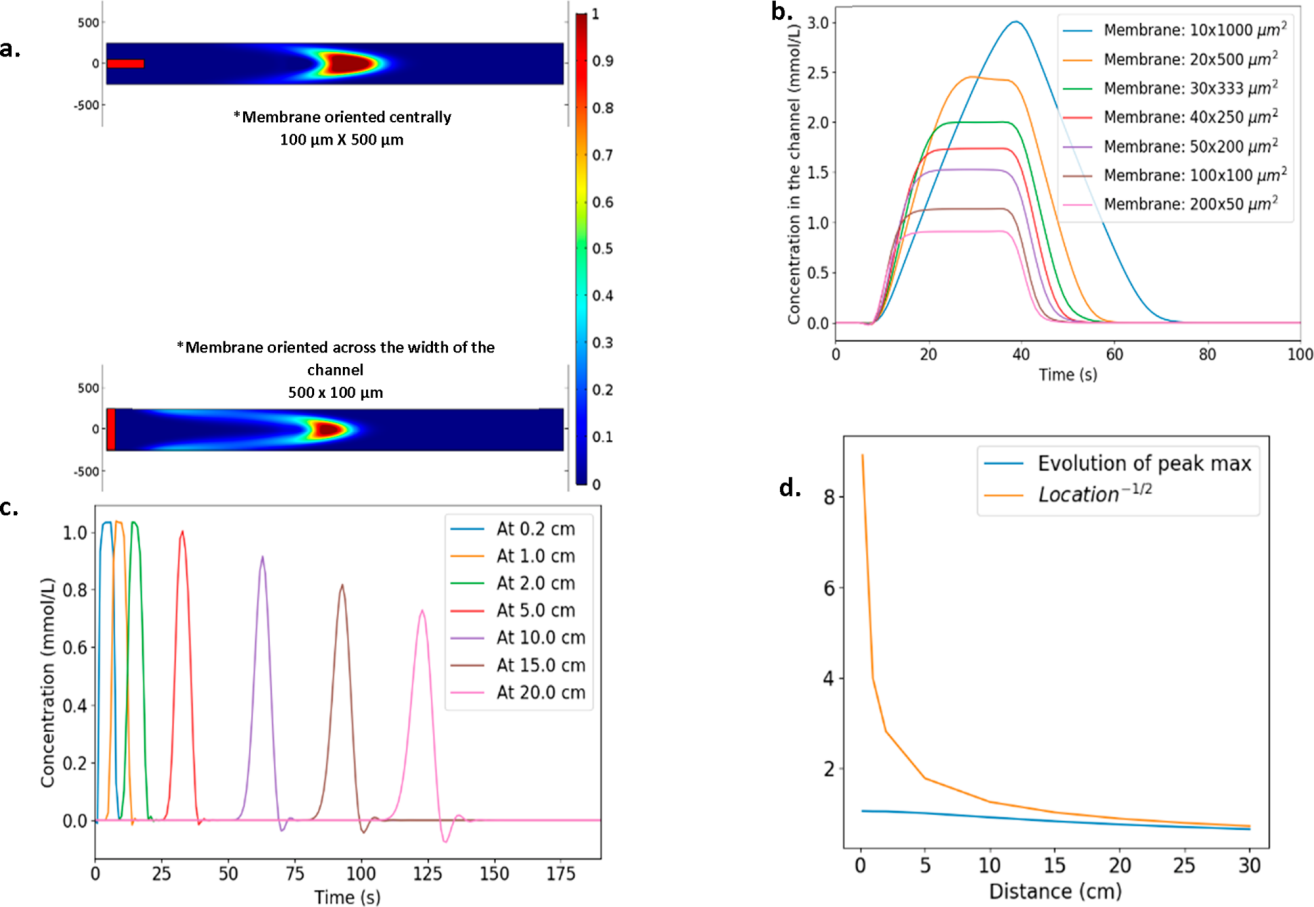
Plots detailing the effect of membrane area, orientation, and the channel length on the concentration profile: (a) Shows two concentration profiles where the orientation of the membrane is central within the channel or spans the channel. Ch_w_ = 500 μm. (b) Shows various plots of the concentration in the channel over time for various different membrane areas. Legend shows width and length of the membrane. (c) Plot of the evolution of a 30 s plateau signal at varying locations of measurement (d) A comparison between the evolution of the peaks shown in Figure 4c, and the predicted evolution was made using the Taylor-Aris equation.

Due to the position of the membrane on one side of the channel only, the channel width and height do not play the same role on the concentration profile within the channel. They do, however, influence both the linear and volume flow rates. Increasing the channel width without increasing the membrane size leads to a more focused diffusion pattern in the longitudinal direction. Particles that cross the membrane are more likely to end up in a part of the channel where, because of the Poiseuille flow pattern, the fluid velocity is, on average higher. Therefore, the difference in velocity between the particles located in the center of the pattern and on the edges of the pattern will be lower, which means that fewer particles are left behind sticking to the walls (bottom profile on Figure 4a).

In Figure 4a, we can see that the configuration with a membrane touching the sides of the channel leads to more drag behind the main body of particles. These particles will be lost below the limit of detection by the time the sensors are reached. The visual analysis of Figure 4a is confirmed by an analysis of concentrations in the channel, as shown in Figure 4b. For a given set of parameters, when the membrane gets longer and narrower, the peak concentration reached in the channel increases. A 50 μm-wide membrane enables a concentration plateau of 1.5 mmol/L, while a 100 μm-wide membrane leads to a lower plateau around 1 mmol/L, despite the membrane areas being equivalent. This is analogous to the increased diffusional flux that is observed with microelectrodes. Results from the model suggest that membranes should be placed within the center of the channel to increase the average velocity of particles in the channel and reduce particle loss at the walls, which reduces the detection of species at the sensors.

#### Channel Length

In the first millimeters of the channel, particles enter the channel through the membrane and are subject to a lot of vertical diffusion, which induces some particle loss. It mainly consists in turning the plateau into a Gaussian curve (first 5 mm on Figure 4c). As a consequence, it is hard to predict the behavior of the peak for short distances.

Afterward, the pattern stabilizes in a Poiseuille flow, which is spread by Taylor-Aris dispersion.

The theoretical expression of Taylor-Aris dispersion is as follows:
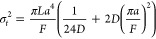
1where σ_t_^2^ is the variance of the Gaussian curve describing the spreading, *L* is the length of the tubing, *a* is the diameter of the tube, *F* is the flow rate, and *D* is the diffusivity of the particles in that fluid.

σ(*L*) = (σ_t_^2^)^1/2^ is a temporal spreading parameter, describing how the resolution of the system evolves.
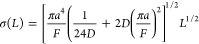
2Using our problem’s variables (channel height Ch_h_, channel width Ch_w_, channel length Ch_l_), let us assume that *a*^2^ ≈ Ch_h_Ch_w_ and *L* = Ch_l_:
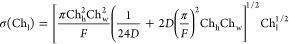
3The height of the Gaussian curve can be expressed as:
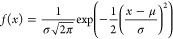
4which means that the peak height, being reached at coordinate μ, is equal to:
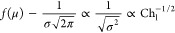
5Therefore, the evolution of the peak height can be related to the evolution of Ch_l_^–1/2^. On Figure 4d, we can see that the further away from the membrane we measure, the closer to an “ideal” behavior the peak is. However, closer to the membrane (≤10 cm), the predicted evolution using the Taylor-Aris equation cannot accurately predict the peak height. Results from the model would suggest that for these types of devices modeling of this nature is highly informative, as there is significant deviation from the predicted behavior of Taylor-Aris dispersion at relevant length scales.

#### Channel height

**Figure 5:**
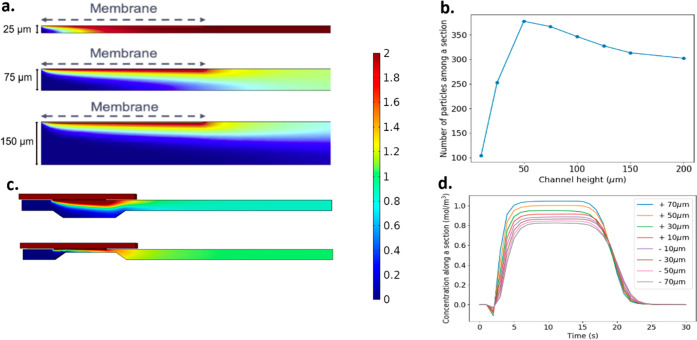
Plots showing the effects of varying channel height and bumps on the concentration profile. (a) Concentration profiles are in the channel section for varying channel heights. (b) A plot shows the number of particles along a section for varying channel heights. The optimum is approximately 200 μm, which is the diffusion length. (c) Two examples of the effect of +ve and −ve bumps on the concentration profile. Ch_w_ = 100 μm, Ch_h_ = 200 μm, Mem_l_ = 200 μm, Mem_w_ = 500 μm, Mem_t_ = 10 μm, tissue is 800 μm by 1100 and 50 μm thick. (d) Plot of the evolution of a 30 s plateau signal for varying +ve and −ve bump heights. (Color scale corresponds to both subfigures a and c).

To be optimal, the channel height needs to be constrained in a small range. When it gets too small, the particles do not have enough depth to diffuse and the cross-membrane gradient stays very low (top of Figure 5a), which impedes more diffusion. It is clear in the figure that the maximum concentration is reached before the end of the membrane, so part of the membrane is effectively not used. On the other hand, when the channel height gets too big, the particles never actually reach the bottom of the channel and are diluted in a larger volume, which decreases the concentration. It can be seen that, at least in this zone of the channel, the whole bottom half of the concentration profile does not carry any particles. It is intuitive that if particles no longer diffuse as the volume increases, the concentration will decrease. As a result, these larger channels are also less efficient in facilitating the entry of particles through the membrane and the concentration detected at the end of the channel.

This can be explained by looking at the velocity profile in a Poiseuille flow. When the channel height increases, the average flow velocity in the diffusion layer decreases. Therefore, particles are washed away less efficiently under the membrane, and the cross-membrane gradient is lower. As a consequence, the particle number decreases when the channel height increases, which leads to an even more diluted signal. Results from the model suggest that for the most efficient delivery of particles and detection, channel height must be found within an optimum range. For small channel heights, the diffusion depth is small, and the cross-membrane gradient is low. For bigger channel heights, the velocity of particles is greatly reduced; therefore, particles are washed away less efficiently, again reducing the cross-membrane gradient. Channel heights that offset both effects are thus the most desirable.

#### Adding Bumps under the Membrane

Deviation from the standard box like channels of these devices could be another way to increase the number of particles that enter the channel and thus the concentration detected at the end of the channel. As discussed before, an increase in the linear velocity helps wash away particles close to the membrane and increases the cross-membrane gradient, but it introduces more volume, which will eventually cause greater dilution. A way to increase the flow without adding dead volume is to add a bump under the membrane. Since the flow is constant, by reducing the channel height, the velocity will increase. We therefore created geometries shown on Figure 5c, with bumps either increasing (+ve bumps) or decreasing (−ve bumps) the velocity. Bumps > 70% of the channel height did not compute.

When more positive bumps were added, an increase in concentration was observed. Even though the effective channel height under the membrane decreases, which could lead to a saturation of the fluid, the acceleration of the fluid decreases the height of the diffusion layer. Therefore, the whole length of the membrane actively picks up particles. As shown in Figure 5d, a +ve bump of 50% of the channel height can lead to an increase of 10% of the concentration in the channel. In addition, the opening out of the channel allows flow-driven mixing into the entire cross section of the channel.

The -ve bumps decrease the velocity under the membrane potentially allowing more time for the particles to diffuse vertically, which could lead to a higher concentration of the fluid. However, Figure 5d shows that the bumps have a negative effect. The likely cause of this is that the beneficial impact of slowing down the fluid is outweighed by the negative effect of adding dead volume.

Results from the model suggest that positive bumps do in fact increase the number of particles that enter the channel and the concentration detected at the end of the channel and can be employed as an alternative to, or in conjunction with, changing the flow rate to increase efficiency of the device.

#### Effects of Bends on the Concentration Profile

**Figure 6:**
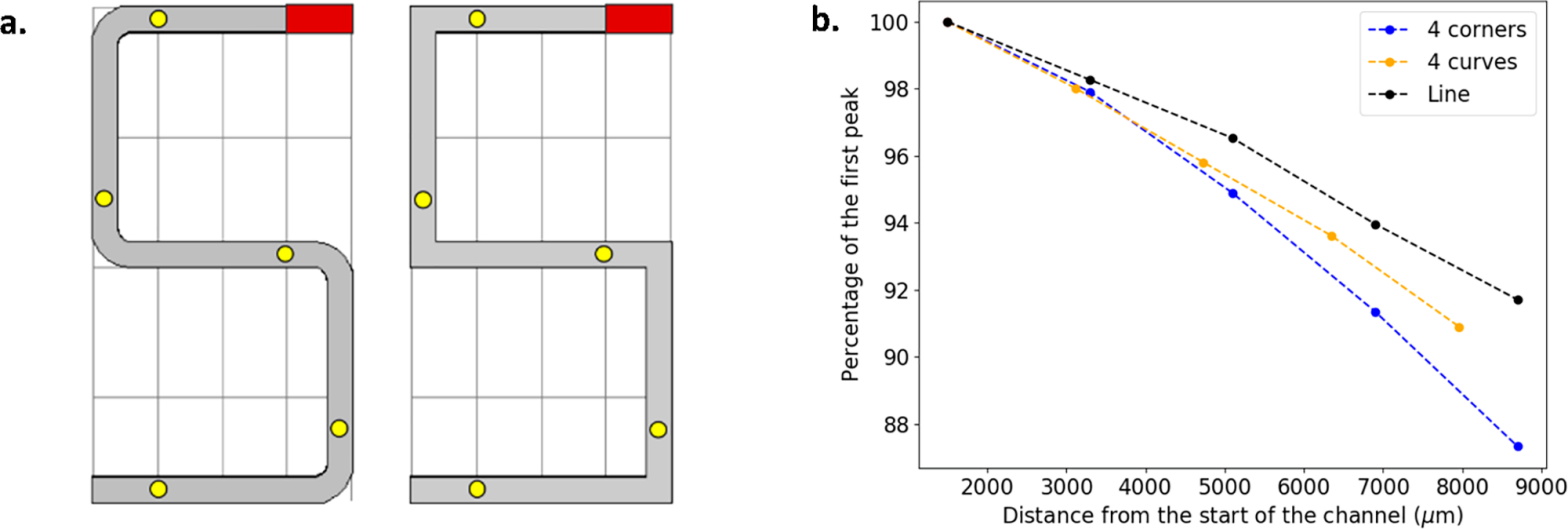
Geometry of channels with corners or curves. The yellow dots on the diagram indicate points of measurement. Ch_w_ = 100 μm, Ch_h_ = 200 μm.

When the channel is changed from a linear geometry to one with corners, some particles are slowed down at the turn and the overall concentration within the channel slightly decreases. In Figure 6b, the black line indicates the decrease in peak height at each measurement point for a straight channel of equivalent length. It can be used as a reference that shows what part of the concentration loss is solely due to dispersion. We then created two S-shaped channels Figure 6a), one with sharp corners (blue line Figure 6b), and one with smooth corners (orange line Figure 6b), in order to assess how the turns can influence the peak height. The measurements are performed at the yellow dots (Figure 6a), so that the influence of each corner could be assessed. Overall, there are as many turns to the right as there are to the left, which can exclude the cumulative effect of losing one side of the concentration profile.

In Figure 6, the blue line is under the orange line, which means that the peak concentration decreases more with corners than with curves. The addition of turns in the geometry leads to the loss of some particles and a decrease of the signal’s peak height. However, smooth curves at these turns perform better than sharp corners at keeping a high concentration. Results from the model suggest that for these devices, channels with linear geometry are the most efficient. When corners and turns are introduced, the resulting signals are reduced but smooth corners are more efficient than sharp corners at limiting particle loss.

### Designing Spatially Independent Membranes

**Figure 7:**
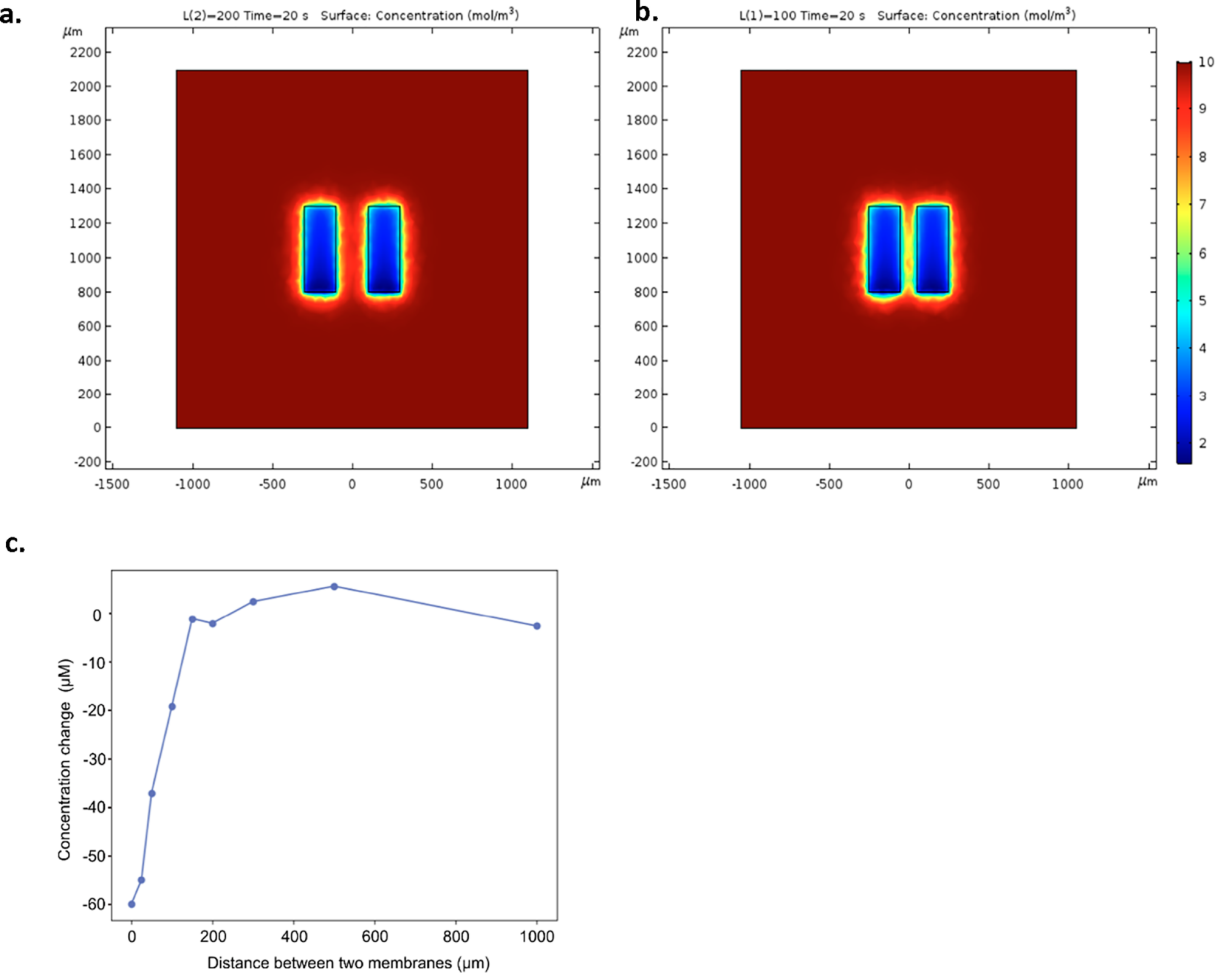
Graphs describing the nature of the diffusion layer around the membrane. The first plot (a) shows two membranes at a distance of 200 μm (left), and the second plot (b) shows two membranes at a distance of 100 μm (right) (Ch_h_ = 100 μm, Ch_w_= 200 μm, Ch_l_= 200 μm, Mem_w_= 200 μm, Mem_l_ = 500 μm, *V* = 25 mm/min). mol/m^3^ = mmol/L. Tissue is 800 μm by 1100 and 50 μm thick. (c) Plot of the concentration change as the distance between the two membranes is increased.

With the development of near-2D chemical sampling probes, the ability to monitor multiple parts of the same tissue in close proximity is desirable. For our intended use case, sampling different parts of a tissue’s chemical profile simultaneously could be useful in the identification of at-risk regions and localizing areas of damage. To demonstrate this, we arranged two equally sized membranes within our models to see the point at which they became ‘independent’ of each other. Figure 7 panels a and b show that at 200 μm there is no interaction, and as the membranes are brought closer to 100 μm there is an overlap between the particles which can be collected at the membrane. This relationship was further illustrated by Figure 7c where the concentration change in micromolar was plotted against the distance between the two membranes in micrometers. At approximately twice the diffusional layer of the tissue, the membranes became independent. Results from the model suggest that membranes as close as 200 μm are independent of each other, and arranging separate channels within this system could facilitate microdialysis measurement in series, an alternative to having to place multiple penetrative microdialysis probes in close proximity.

## Methods

### Design Requirements

**Figure 8:**
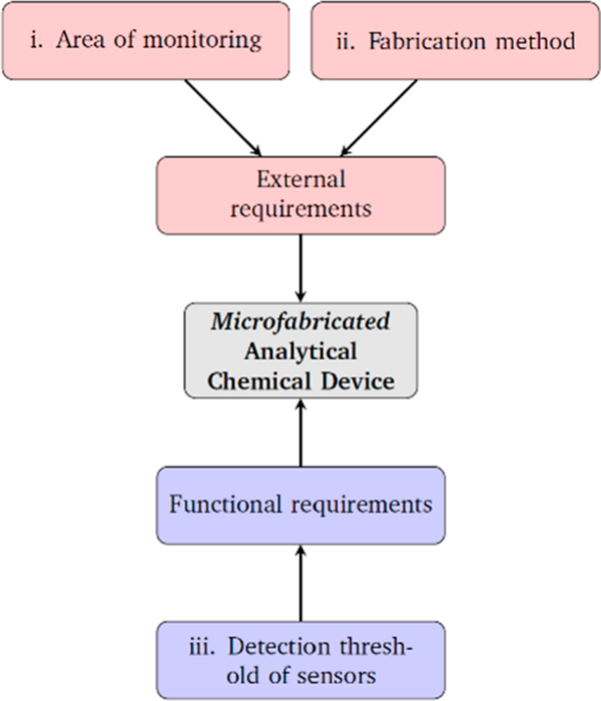
Requirements imposed on our microfabricated analytical chemical device.

The design requirements for a new device are schematically listed in Figure 8. We consider as “external requirements” the type of tissue monitoring required and the limitations of the fabrication methods. The examples shown in this paper relate to subdural monitoring of the human brain, where device thickness is important and multiple sampling sites desirable. For fabrication we consider chemical vapor deposition which can be employed to deposit thin (on the order of a few micrometers), pinhole free layers of soft polymers which can be used to form channels and potential sampling areas.^39^ However, the fabrication processes that create such probes also induce geometric constraints.

Functional requirements also guided the development of our models. Desiring the detection of micromolar dynamic concentration changes requires high efficiency of molecular pick-up by the sampling element coupled to minimal dispersion before reaching the detection sensor. This gives the best chance of exceeding the limit of detection of the sensor. Low dispersion also allows for effective separation of consecutive signals. These requirements led to the generation of a range of parameters, which are fully outlined in Figure 10b. These parameters defined the nature of the channel, membrane, and the motion of molecules within these two structures.

### Applicability to the Brain

**Figure 9:**
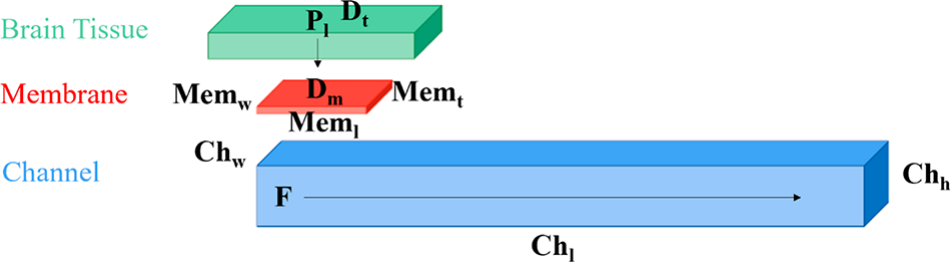
Components of the 3D model with labeled parameters that correspond to the brain tissue (green), membrane (red), and channel (blue): pulse length (P_l_), diffusion of the tissue (D_t_), membrane width (Mem_w_), membrane length (Mem_l_), membrane thickness (Mem_t_), channel width (Ch_w_), channel length (Ch_l_), channel height (Ch_h_), and flow (F).

Within our modeling, we set any applicable parameters to resemble values that we would expect to see in the brain. The diffusion that occurred within the tissue was therefore modeled as brain tissue where effects such as tortuosity were considered. Tortuosity of a tissue is defined by the equation λ = *D*/*D*_t_, where *D* is the diffusion coefficient in an obstacle free medium and *D*_t_ is the diffusion coefficient in the tissue. It summarizes how the structure and connectivity of a tissue limits diffusion. In normal brain tissue, λ = 1.44, while in ischemic tissue, this can rise to approximately 2.2 (higher for larger molecules).^40,41^ As a result, we assumed that 1.10^–10^ m^2^ s^–1^ < *D*_t_ < 5.10^–10^ m^2^ s^–1^ We also set minimum channel lengths so that sensors were not placed too close to the brain to potentially introduce infection or toxicity.^42^ We also constrained the maximum volume of the system, as we wanted a device that would have as small a footprint as possible with a view to implantation. This led to a model that had defined parameters for the brain tissue, in addition to the membrane and channel, as seen in Figure 9.

### Parameter Range

**Figure 10:**
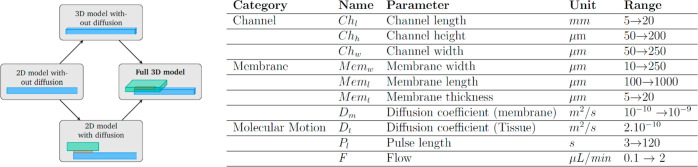
Design flow and parameters used to develop complex, informative models: Shows the process of first developing a 2D model without diffusion where the signal emanates from the channel boundary, this is followed by the development of a 3D model with similar characteristics and a 2D model with a membrane and tissue. These two subsequent models are then combined to create a full 3D model. (The channel is blue, membrane red, and tissue green.) (b) The parameters used to adjust the models created. Parameters pertained to properties of the channel, membrane, and molecular motion and had different units and constrained ranges.

Further consideration of our external and functional requirements led us to define restricted ranges for each parameter of interest. These ranges, alongside a full list of parameters, are outlined in Figure 10. This helped limit the computational time for simulations and created a model that more realistically mirrors the experimental world.

### Computational Fluid Modeling Method

To evaluate the concentration gradients in the channel, the flow was modeled using COMSOL Multiphysics 5.5 (COMSOL Ltd., Cambridge, UK), with primary focus on the laminar flow and particle diffusion modules. The design was drawn directly in COMSOL. The Transport of Diluted Species (tds) interface, a predefined modeling environment for investigating the evolution of chemical species, was used to calculate the concentration field of a dilute solute in a solvent. The driving force for transport in this case can be diffusion by Fick’s law, convection when a flow field is present, or migration when an electric field is applied. In our case, the solute diffuses through the tissue and membrane, abiding by Fick’s laws and is then subject to diffusion and convection within the channel. The interface assumes that all species present have a small concentration when compared to the solute; therefore, mixture properties such as density and viscosity are assumed to be dictated by the solvent. The simulation was also performed by using the steady state Navier–Stokes equation. The inlet and outlet ports were specified at the beginning and end of the geometry. The parameters listed in Figure 10b were adjusted for each simulation. All meshes were generated automatically by COMSOL, using the “Physics-controlled” option. For most simulations, “coarse” or “coarser” meshes were enough to achieve convergence of the computation and to avoid singularities, while keeping the computation time under a reasonable limit. If necessary, “normal” or “fine” meshes were implemented. Results were analyzed initially within the software using multiphysics studies on surface and section plots, followed by exporting raw concentration data along sections and/or with time with Python post-processing.

### Increasing Complexity

The model was constructed by assembling several basic sections and iteratively adding complexity (Figure 10a). Initially, 2D simulations, made of a simple channel with an input signal at a boundary, were created. They were used to understand the phenomenon of Taylor dispersion and test Ch_w_ and Ch_h_ parameter changes. Boundary conditions were also tested at this stage, by creating different tissue sizes, to analyze the effect of open boundaries. 3D models based on the same 2D structure were then created. In conjunction, diffusion through a membrane of varying thickness and diffusion coefficient was first studied in a static model and then added to a channel with laminar flow circulation. Eventually, all of the models converged to a full 3D model: with a simulated tissue, a membrane, and a channel with laminar flow.

## Conclusions

Overall, we have shown a range of considerations that must be taken into account when designing a near-2D sampling probe. The use of COMSOL modeling has informed the optimum ranges for channel heights, widths, and lengths, membrane geometries, and the introduction of corners, bends, and bumps. Not all of these features can be reproduced easily by experimental fabrication processes, but they serve as a very useful guide to steer the creation of an efficient chemical sampling probe that is nonpenetrative in nature.

From the modeling demonstrated here, a set of ideal characteristics have been identified that can be translated to physical experiments. Examples include: channel geometry being constructed as linearly as possible to reduce particle loss, the membrane being situated centrally within the channel, avoiding the edges, and the membrane being extended or better yet segmented to improve the number of particles that enter the channel and ultimately boost the concentration that is detected at the end of the channel. The information gleaned from this modeling will directly feed into the development of flexible, near 2D probes that can accurately and efficiently sample bodily tissue with a comparative and hopefully improved performance (with respect to relative recovery) to conventional, concentric microdialysis probes without creating further implantation injuries.

## References

[ref1] Booth M. A.; Gowers S. A.; Leong C. L.; Rogers M. L.; Samper I. C.; Wickham A. P.; Boutelle M. G. Anal. Chem. 2018, 90, 2–18. 10.1021/acs.analchem.7b04224. 29083872

[ref2] Broza Y. Y.; Zhou X.; Yuan M.; Qu D.; Zheng Y.; Vishinkin R.; Khatib M.; Wu W.; Haick H. Chem. Rev. 2019, 119, 11761–11817. 10.1021/acs.chemrev.9b00437. 31729868

[ref3] Litchfield I.; Bentham L.; Hill A.; McManus R. J.; Lilford R.; Greenfield S. BMJ. Qual. Saf. 2015, 24, 681–690. 10.1136/bmjqs-2014-003690. PMC4680130 26251507

[ref4] Samper I. C.; Gowers S. A.; Rogers M. L.; Murray D. S. R.; Jewell S. L.; Pahl C.; Strong A. J.; Boutelle M. G. Lab Chip 2019, 19, 2038–2048. 10.1039/C9LC00044E. 31094398 PMC9209945

[ref5] Frost M. C.; Meyerhoff M. E. Annu. Rev. Anal. Chem. 2015, 8, 171–192. 10.1146/annurev-anchem-071114-040443. 26161973

[ref6] Zimphango C.; Alimagham F. C.; Carpenter K. L. H.; Hutchinson P. J.; Hutter T. Metabolites 2022, 12 (5), 393 10.3390/metabo12050393. 35629896 PMC9146878

[ref7] Alimagham F. C.; Hutter D.; Marco-Garcia N.; Gould E.; Highland V. H.; Huefner A.; Giorgi-Coll S.; Killen M. J.; Zakrzewska A. P.; Elliott S. R.; Carpenter K. L. H.; Hutchinson P. J.; Hutter T. Anal. Chem. 2021, 93 (35), 11929–11936. 10.1021/acs.analchem.1c01149. 34432431 PMC8427560

[ref8] Kozai T. D.; Catt K.; Li X.; Gugel Z. V.; Olafsson V. T.; Vazquez A. L.; Cui X. T. Biomaterials 2015, 37, 25–39. 10.1016/j.biomaterials.2014.10.040. 25453935 PMC4312222

[ref9] Delbeck S.; Vahlsing T.; Leonhardt S.; Steiner G.; Heise H. M. Anal. Bioanal. Chem. 2019, 411, 63–77. 10.1007/s00216-018-1395-x. 30283998

[ref10] Gowers S. A.; Curto V. F.; Seneci C. A.; Wang C.; Anas- tasova S.; Vadgama P.; Yang G. Z.; Boutelle M. G. Anal. Chem. 2015, 87, 7763–7770. 10.1021/acs.analchem.5b01353. 26070023 PMC4526885

[ref11] Rogers M. L; Leong C. L.; Gowers S. A.; Samper I. C; Jewell S. L; Khan A.; McCarthy L.; Pahl C.; Tolias C. M; Walsh D. C; Strong A. J; Boutelle M. G Blood Flow Metab. 2017, 37, 1883–1895. 10.1177/0271678X16674486. PMC5414898 27798268

[ref12] Gowers S. A.; Rogers M. L.; Booth M. A.; Leong C. L.; Samper I. C.; Phairatana T.; Jewell S. L.; Pahl C.; Strong A. J.; Boutelle M. G. Lab Chip 2019, 19, 2537–2548. 10.1039/C9LC00400A. 31290529 PMC7321805

[ref13] Rogers M. L.; Feuerstein D.; Leong C. L.; Takagaki M.; Niu X.; Graf R.; Boutelle M. G. ACS Chem. Neurosci. 2013, 4, 799–807. 10.1021/cn400047x. 23574576 PMC3656742

[ref14] Gowers S. A.; Samper I. C.; Murray D. S. R.; Smith G. K.; Jeyaprakash S.; Rogers M. L.; Karlsson M.; Olsen M. H.; Moller K.; Boutelle M. G. Analyst 2020, 145, 1894–1902. 10.1039/C9AN01950B. 31984382

[ref15] Hutchinson P. J.; Jalloh I.; Helmy A.; Carpenter K. L.; Rostami E.; Bellander B. M.; Boutelle M. G.; Chen J. W.; Claassen J.; Dahyot-Fizelier C.; Enblad P.; Gallagher C. N.; Helbok R.; Hillered L.; Le Roux P. D.; Magnoni S.; Mangat H. S.; Menon D. K.; Nordström C. H.; O’Phelan K. H.; Oddo M.; Barcena J. Perez; Robertson C.; Ronne-Engström E.; Sahuquillo J.; Smith M.; Stocchetti N.; Belli A.; Carpenter T. A.; Coles J. P.; Czosnyka M.; Dizdar N.; Goodman J. C.; Gupta A. K.; Nielsen T. H.; Marklund N.; Montcriol A.; O’Connell M. T.; Poca M. A.; Sarrafzadeh A.; Shannon R. J.; Skjo̷th-Rasmussen J.; Smielewski P.; Stover J. F.; Timofeev I.; Vespa P.; Zavala E.; Ungerstedt U. Intensive Care Med. 2015, 41, 1517–1528. 10.1007/s00134-015-3930-y. 26194024 PMC4550654

[ref16] Groth L.; Serup J. Acta Derm. Venereol. 1998, 78, 5–9. 10.1080/00015559850135733. 9498017

[ref17] Varner E. L.; Leong C. L.; Jaquins-Gerstl A.; Nesbitt K. M.; Boutelle M. G.; Michael A. C. ACS Chem. Neurosci. 2017, 8, 1779–1788. 10.1021/acschemneuro.7b00148. 28482157 PMC6677237

[ref18] Robbins E. M.; Jaquins-Gerstl A.; Fine D. F.; Leong C. L.; Dixon C. E.; Wagner A. K.; Boutelle M. G.; Michael A. C. ACS chemical neuroscience 2019, 10, 3521–3531. 10.1021/acschemneuro.9b00145. 31246409 PMC7341684

[ref19] Stocchetti N.; Roux P.; Vespa P.; Oddo M.; Citerio G.; Andrews P. J; Stevens R. D; Sharshar T.; Taccone F. S; Vincent J.-L. Crit Care 2013, 17, 201 10.1186/cc11513. 23320763 PMC4057243

[ref20] Cho K. W.; Sunwoo S.; Hong Y. J.; Koo J. H.; Kim J. H.; Baik S.; Hyeon T.; Kim D. Chem. Rev. 2022, 122 (5), 5068–5143. 10.1021/acs.chemrev.1c00531. 34962131

[ref21] Abrahamsson P.; Aberg A.; Winso O.; Johansson G.; Haney M.; Blind P. Clin. Phys. and Funct. Imag. 2012, 32 (2), 99–105. 10.1111/j.1475-097X.2011.01061.x. 22296629

[ref22] Akesson O.; Abrahamsson P.; Johansson G.; Blind P. J. Surg Res. 2016, 204, 39 10.1016/j.jss.2016.04.001. 27451866

[ref23] Abrahamsson P.; Aberg A.-M.; Johansson G.; Winso O.; Waldenstrom A.; Haney M. Clin. Phys. and Funct. Imag. 2011, 31 (3), 175–181. 10.1111/j.1475-097X.2010.00995.x. 21091606

[ref24] Ronaldson-Bouchard K.; Vunjak-Novakovic G. Cell stem cell 2018, 22, 310–324. 10.1016/j.stem.2018.02.011. 29499151 PMC5837068

[ref25] Wikswo J. P.; Block F. E. III; Cliffel D. E.; Goodwin C. R.; Marasco C. C.; Markov D. A.; McLean D. L.; McLean J. A.; McKenzie J. R.; Reiserer R. S.; et al. IEEE Transactions on Biomedical Engineering 2013, 60, 682–690. 10.1109/TBME.2013.2244891. 23380852 PMC3696887

[ref26] Samper I. C.; Gowers S. A.; Booth M. A.; Wang C.; Watts T.; Phairatana T.; Vallant N.; Sandhu B.; Papalois V.; Boutelle M. G. Anal. Chem. 2019, 91, 14631 10.1021/acs.analchem.9b03774. 31647870 PMC7110273

[ref27] Zhou Q.; Patel D.; Kwa T.; Haque A.; Matharu Z.; Stybayeva G.; Gao Y.; Diehl A. M.; Revzin A. Lab Chip 2015, 15, 4467–4478. 10.1039/C5LC00874C. 26480303

[ref28] Wu J.; Dong M.; Rigatto C.; Liu Y.; Lin F. npj Digit. Med. 2018, 1, 1–11. 10.1038/s41746-017-0014-0. 31304292 PMC6550168

[ref29] Sala-Jarque J.; Mesquida-Veny F.; Badiola-Mateos M.; Samitier J.; Hervera A.; del Rio J. A. Cells 2020, 9, 302 10.3390/cells9020302. 32012727 PMC7072511

[ref30] Jin H.; Abu-Raya Y. S.; Haick H. Adv. Healthc. Mater. 2017, 6, 11 10.1002/adhm.201700024. 28371294

[ref31] Carvalho V.; Rodrigues R. O.; Lima R. A.; Teixeira S. Micromachines 2021, 12 (10), 1149 10.3390/mi12101149. 34683199 PMC8539624

[ref32] Rognin E.; Willis-Fox N.; Daly R. Sci. Rep 2023, 13, 1900 10.1038/s41598-023-28915-3. 36732612 PMC9894834

[ref33] Cepeda D. E.; Hains L.; Li D.; Bull J.; Lentz S. I.; Kennedy R. T. J. Neurosci Methods. 2015, 242, 97–105. 10.1016/j.jneumeth.2015.01.019. 25614385 PMC4331210

[ref34] Lee W. H.; Slaney T. R.; Hower R. W.; Kennedy R. T. Anal. Chem. 2013, 85 (8), 3828–3831. 10.1021/ac400579x. 23547793 PMC3642770

[ref35] Bakuova N.; Toktarkan S.; Dyussembinov D.; Azhibek D.; Rakhymzhanov A.; Kostas K.; Kulsharova G. Biosensors 2023, 13 (7), 754 10.3390/bios13070754. 37504152 PMC10377015

[ref36] Introduction to COMSOL Multiphysics: Comsol, 1998.

[ref37] McIntyre D.; Lashkaripour A.; Fordyce P.; Densmore D. Lab Chip 2022, 22, 2925–2937. 10.1039/D2LC00254J. 35904162 PMC9361804

[ref38] Maionchi D. d. O.; Ainstein L.; dos Santos F. P.; de Souza Junior M. B. Int. J. Heat Mass Transfer 2022, 194, 123110 10.1016/j.ijheatmasstransfer.2022.123110.

[ref39] Tan C. P.; Craighead H. G. Materials (Basel). 2010, 3, 1803–1832. 10.3390/ma3031803.

[ref40] Syková E.; Nicholson C. Physiol. Rev. 2008, 88, 1277–1340. 10.1152/physrev.00027.2007. 18923183 PMC2785730

[ref41] Endocrine T. C.; Res J. L. F. C.; Moss R.; Nicholson C.; Syková E.; Nicholson C. Essentials of Glycobiology 1996, 207–215.

[ref42] Van Epps J. S.; Younger J. G. Shock 2016, 46, 597–608. 10.1097/SHK.0000000000000692. 27454373 PMC5110396

